# SUMOylation of FOXM1B Alters Its Transcriptional Activity on Regulation of MiR-200 Family and JNK1 in MCF7 Human Breast Cancer Cells

**DOI:** 10.3390/ijms150610233

**Published:** 2014-06-10

**Authors:** Chiung-Min Wang, Runhua Liu, Lizhong Wang, Leticia Nascimento, Victoria C. Brennan, Wei-Hsiung Yang

**Affiliations:** 1 Department of Biomedical Sciences, Mercer University School of Medicine, Savannah, GA 31404, USA; E-Mails: meowy200@yahoo.com (C.-M.W.); ln6739@stu.armstrong.edu (L.N.); v.brennan@umiami.edu (V.C.B.); 2 Department of Genetics and Comprehensive Cancer Center, University of Alabama at Birmingham, Birmingham, AL 35294, USA; E-Mails: runhua@uab.edu (R.L.); lwang12@uab.edu (L.W.)

**Keywords:** FOXM1, SUMOylation, transcriptional activity, *MiR-200b/c*, *JNK1*

## Abstract

Transcription factor Forkhead Box Protein M1 (FOXM1) is a well-known master regulator in controlling cell-cycle pathways essential for DNA replication and mitosis, as well as cell proliferation. Among the three major isoforms of FOXM1, FOXM1B is highly associated with tumor growth and metastasis. The activities of FOXM1B are modulated by post-translational modifications (PTMs), such as phosphorylation, but whether it is modified by small ubiquitin-related modifier (SUMO) remains unknown. The aim of the current study was to determine whether FOXM1B is post-translationally modified by SUMO proteins and also to identify SUMOylation of FOXM1B on its target gene transcription activity. Here we report that FOXM1B is clearly defined as a SUMO target protein at the cellular levels. Moreover, a SUMOylation protease, SENP2, significantly decreased SUMOylation of FOXM1B. Notably, FOXM1B is selectively SUMOylated at lysine residue 463. While SUMOylation of FOXM1B is required for full repression of its target genes *MiR-200b/c* and *p21*, SUMOylation of FOXM1B is essential for full activation of *JNK1* gene. Overall, we provide evidence that FOXM1B is post-translationally modified by SUMO and SUMOylation of FOXM1B plays a functional role in regulation of its target gene activities.

## 1. Introduction

Forkhead Box Proteins (FOX proteins) are transcription factors consisting of more than 55 mammalian proteins and sharing a 100-amino acid long, evolutionarily conserved winged helix DNA-binding region [1]. FOXM1 (previously known as FKHL16, HFH11, and MPP2) plays a crucial role in the regulation of cell cycle progression and cell proliferation [2,3,4,5]. FOXM1 is expressed in all replicating cells, but not in quiescent and terminally differentiated cells. FOXM1 is regulated by numerous oncogenic signals, growth factors, p53, pRb, p19ARF, and itself (by auto-regulation) [6,7,8,9,10,11]. As a transcription factor, FOXM1 targets and controls a variety of genes, including *CTNNB1* [12,13,14] for adherens junctions and cell self-renewal, *CDKN1A* [15] for cell proliferation, *VEGF* [16] for blood vessel formation, *MMP2* [17] and *JNK1* [18] for cell migration, *HELLS* [19] and *SKP2* [20] for cell cycle regulation, and *NR3A1* [21] for estrogen signaling in humans by binding to promoter regions with a preference for a conserved consensus 5'-TAAACA-3' sequence. Several lines of evidence have demonstrated that FOXM1 is associated with tumor initiation, promotion, invasion, and metastasis, suggesting that FOXM1 contributes to all major hallmarks of cancer [22]. Studies have confirmed that FOXM1 expression levels correlate with poor prognosis [23,24]. Moreover, amplifications of *FOXM1* gene have been demonstrated in several tumors such as hepatocellular cancer, pancreatic cancer, and glioblastoma multiforme tumors [13,25,26,27]. Therefore, targeting FOXM1 (the relay center for cancer development and a potential prognostic marker) holds a promising therapeutic intervention.

The majority of the transcription factors are functionally regulated by post-translational modifications (PTMs) which are essential for normal physiological functions in cells and efficient ways for the cells to respond to multiple extra-cellular stimuli and intra-cellular signals. Among the various post-translational modifications, the modification by small ubiquitin-related modifier (SUMO) family has profound effects on regulating normal cell physiology and tumorigenesis [28,29,30,31,32,33,34]. In spite of limited sequence identity, SUMO proteins are structurally related to ubiquitin and use a similar three-step enzyme-controlled cascade reaction. The carboxyl-terminal glycine in the processed SUMO protein covalently binds to an internal lysine residue of the target protein. Importantly, covalent conjugation of proteins by SUMO is highly transient, dynamic, and reversible through action of the SENP family of proteases. In normal cellular conditions, less than 5% of the target proteins will be SUMOylated [35]. Even though the three-dimensional structure and conjugation mechanism of SUMO share similarities to those of ubiquitin, the biological functions of SUMOylation are significantly different from those of ubiquitination [35]. SUMOylation mainly prevents ubiquitin-mediated proteasomal protein degradation and usually enhances protein stability [35,36]. Majority of the SUMO substrates are transcription factors and co-factors. Most importantly, SUMO modification of transcription factors and nuclear receptors has a strong impact on their regulation of transcription of genes [33,37,38,39,40], such as SUMO1 modification activates the transcriptional response of p53 [41] and SUMOylation inhibits NR5A1 activity [33]. Several components of the SUMO pathway, such as UBE2I (the only E2-conjugating enzyme for SUMOylation) [42,43] and protein inhibitor of activated STAT (PIAS) proteins [44], are also involved in regulation of transcription. In this regard, understanding the regulation of SUMO processes is vital for various biological processes such as the regulation of transcription and the development of disorders. Therefore, the manipulation of SUMO modification and processes has gained attention as a potential therapeutic intervention.

Accumulated evidence indicates that PTMs regulate FOXM1 functions. For example, during the cell cycle progression, FOXM1 expression is markedly elevated at the G_1_/S and G_2_/M transition and multisite phosphorylations on FOXM1 by various kinases (such as MAPK, CDKs, and PLK1) are essential for FOXM1 activity for mitotic entry and progression, ensuring the genomic stability [45,46,47,48]. Alternative splicing of *FOXM1* gene gives rise to three major isoforms of FOXM1, the transcriptionally inactive FOXM1A, and transcriptionally active FOXM1B and FOXM1C variants [49]. Extensive studies have shown that FOXM1B is the predominant isoform that is over-expressed in most human cancers and exhibits a higher transforming ability than FOXM1C, the canonical form in most normal cells [10,17,50,51,52]. Moreover, FOXM1B has been demonstrated to be a potent activator of tumor metastasis [53]. Therefore, we chose FOXM1B as a desirable target to study whether SUMOylation influences FOXM1B transcriptional activity in MCF7 human breast cancer cells. In this study, we demonstrated that FOXM1B is a substrate for SUMO modification and FOXM1B transcriptional activity requires conjugating of SUMO to mediate efficient SUMOylation of FOXM1B at lysine 463.

## 2. Results

### 2.1. Forkhead Box Protein M1 B (FOXM1B) Is a Substrate for Modification by Small Ubiquitin-Related Modifier (SUMO)

**Figure 1:**
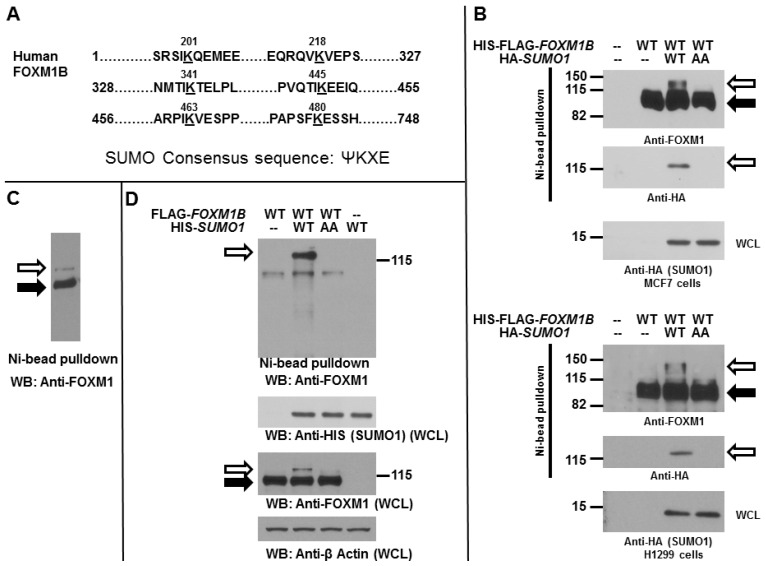
FOXM1B can be SUMOylated. (**A**) Sequence of the human FOXM1B protein showing the regions that contain the potential SUMO sites (K201, K218, K341, K445, K463, and K480). Abbreviations: FOXM1, Forkhead Box Protein M1; SUMO, small ubiquitin-related modifier; (**B**) MCF7 (**upper**) and H1299 (**bottom**) cells were transiently transfected with 3 µg HIS-FLAG-tagged WT *FOXM1B* and 2 µg HA-*SUMO1* (WT or AA mutant) expression plasmids as indicated. After 48 h, cells were harvested and the cell lysates were subjected to Ni^2+^ bead pulldown, followed by anti-FOXM1 or anti-HA immunoblotting. Whole cell lysates (WCL) were subjected to anti-HA immunoblotting for SUMO1 expression. The empty arrows indicate SUMOylated FOXM1B; The solid arrows indicate non-SUMOylated FOXM1B; (**C**) MCF7 cells were transiently transfected with 3 µg HIS-tagged WT *FOXM1B* expression plasmids. After 48 h, cells were harvested and the cell lysates were subjected to Ni^2+^ bead pulldown, followed by anti-FOXM1 immunoblotting. The empty arrows indicate SUMOylated FOXM1B; The solid arrows indicate non-SUMOylated FOXM1B; (**D**) MCF7 cells were transiently transfected with 2 µg FLAG-tagged WT *FOXM1B* and 2 µg HIS-*SUMO1* (WT or AA mutant) expression plasmids as indicated. After 48 h, cells were harvested and the cell lysates were subjected to Ni^2+^ bead pulldown, followed by anti-FOXM1 immunoblotting. WCL were subjected to anti-HA, anti-FOXM1, or anti-β-Actin immunoblotting for SUMO1, FOXM1B, or β-Actin expression, respectively. The empty arrows indicate SUMOylated FOXM1B; The solid arrows indicate non-SUMOylated FOXM1B.

Human FOXM1B protein harbors several evolutionarily conserved sequences that conform to the typical SUMOylation consensus (Figure 1A). To determine whether FOXM1B can be SUMOylated by SUMO1 in mammalian cells, MCF7 breast cancer and H1299 lung cancer cells were transiently transfected with HIS-FLAG tagged *FOXM1B* expression plasmids with or without HA-tagged *SUMO1* (WT or AA mutant) expression plasmids. Western blot analysis (Figure 1B) of the FOXM1B preparations by Ni^2+^ chelate chromatography under denaturing conditions, revealed that a slowly migrating species (about 120–130 kDa) was detected in cells expressing WT FOXM1B and WT SUMO1. However, AA mutant SUMO1, of which the *C*-terminal di-glycine residues (which are required for SUMO conjugation to substrates) of SUMO1 were mutated to di-alanine residues, could not increase FOXM1B SUMOylation. We next investigated whether FOXM1B can be SUMOylated endogenously in MCF7 cells. As shown in Figure 1C, when MCF7 cells were over-expressed with HIS-tagged *FOXM1B* expression plasmids, western blot analysis of the FOXM1B preparations by Ni^2+^ chelate chromatography under denaturing conditions revealed that a slowly migrating species (about 120–130 kDa) was detected in cells, suggesting that FOXM1B can be endogenously modified by SUMO in cells. During 10 times of the experiments, we observed that FOXM1B can be endogenously SUMOylated five times. To further confirm the previous results (Figure 1B), we expressed FLAG-tagged *FOXM1B* with HIS-tagged WT *SUMO1* or AA *SUMO1* mutant in MCF7 cells (Figure 1D). The SUMOylated proteins were purified using Ni^2+^–NTA resins under denaturing conditions. The SUMOylation of FOXM1B was detected by using an anti-FOXM1 antibody. Our data showed that the SUMOylated FOXM1B was clearly detected with a slower migrating band with molecular weight greater than 120 kDa in sodium dodecyl sulfate-polyacrylamide gel electrophoresis (SDS-PAGE) gel (Figure 1C). In contrast, AA SUMO1 mutant completely lost the ability to promote FOXM1B SUMOylation (Figure 1C). These results indicate that band shift of FOXM1B was indeed due to the covalent conjugation of SUMO. Overall, our data showed that FOXM1B is a target of SUMOylation.

### 2.2. SUMOylation of FOXM1B Is Modulated by SENP2 and PIASy

**Figure 2:**
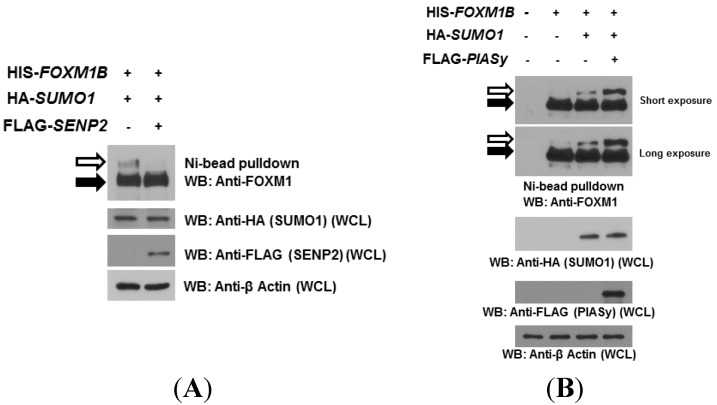
SENP2 reduces and PIASy enhances FOXM1B SUMOylation. (**A**) MCF7 cells were co-transfected HIS-*FOXM1B* and HA-*SUMO1*with or without FLAG-tagged *SENP2* plasmids. Forty-eight hours later, cell lysates were subjected to Ni^2+^ bead pulldown, followed by anti-FOXM1 immunoblotting. Whole cell lysates (WCL) were subjected to anti-HA, anti-FLAG, or anti-β Actin immunoblotting for SUMO1, SENP2, or β-Actin expression, respectively. The empty arrows indicate SUMOylated FOXM1B; The solid arrows indicate non-SUMOylated FOXM1B; (**B**) MCF7 cells were co-transfected HIS-*FOXM1B* and HA-*SUMO1* with or without FLAG-tagged *PIASy* plasmids. Forty-eight hours later, cell lysates were subjected to Ni^2+^ bead pulldown, followed by anti-FOXM1 immunoblotting. Whole cell lysates (WCL) were subjected to anti-HA, anti-FLAG, or anti-β Actin immunoblotting for SUMO1, PIASy, or β-Actin expression, respectively. The empty arrows indicate SUMOylated FOXM1B; The solid arrows indicate non-SUMOylated FOXM1B.

Generally, SENP proteins are responsible for activating and de-conjugating SUMO from target proteins. In particular, SENP1 and SENP2 participate in this de-conjugation in mammals. Thus, we next examined whether SENP2 de-SUMOlates FOXM1B. We expressed HIS-tagged *FOXM1B* and HA-tagged *SUMO1* with or without FLAG-tagged *SENP2* in MCF7 cells. As shown in Figure 2A, a SUMOylated FOXM1B band was observed in cells expressing FOXM1B and SUMO1. However, when SENP2 was co-expressed with FOXM1B and SUMO1 in cells, the SUMOylated band was completely lost, suggesting that SENP2 was involved in mediating the de-SUMOylation of FOXM1B.Many studies have shown that E3 ligases, such as PIAS proteins, in the SUMOylation cycle function as adaptors and facilitators that stabilize the interaction between the SUMO-UBE2I thioester and the acceptor substrates. Therefore, we next investigated whether PIASy, one of PIAS family of proteins, is capable of facilitating FOXM1B SUMOylation in MCF7 cells. When cells were transiently expressed FOXM1B alone, the SUMOylated FOXM1B band was observable (long exposure, Figure 2B), suggesting that FOXM1B is capable to be SUMOylated endogenously (consistent with the result of Figure 1C). When SUMO1 was co-expressed with FOXM1B, the intensities of the SUMOylated FOXM1B bands were increased as expected. Interestingly, the intensities of the SUMOylated FOXM1B bands were further significantly increased when PIASy was co-expressed with FOXM1B and SUMO1, suggesting that PIASy enhances the SUMOylation of FOXM1B.

### 2.3. Lysine 463 Is the Major SUMO Site in FOXM1B

**Figure 3:**
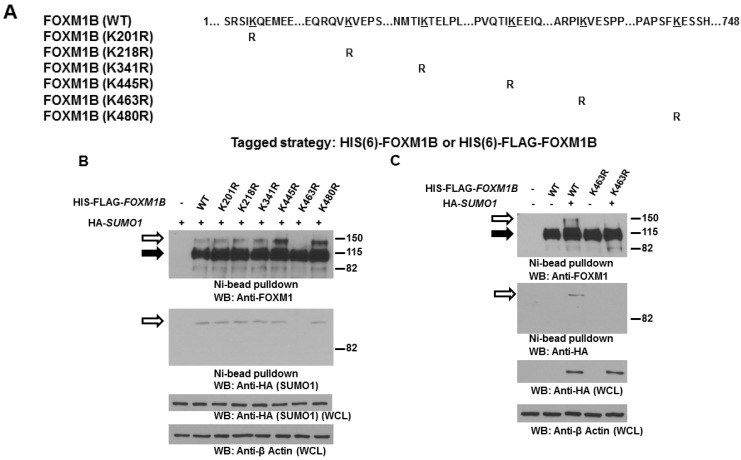
Lysine 463 is the major SUMO site in FOXM1B. (**A**) Schematic representation of the human FOXM1B protein with the lysine-to-arginine FOXM1B mutants generated in this study to determine potential SUMOylation sites on FOXM1B; (**B**) Lysates of MCF7 cells transiently transfected with 3 µg HIS-FLAG tagged WT or mutant *FOXM1B* expression plasmid and 1 µg HA-*SUMO1* expression plasmid were subjected to Ni^2+^ bead pulldown, followed by anti-FOXM1 and anti-HA immunoblotting. The empty arrows indicate SUMOylated FOXM1B; The solid arrows indicate non-SUMOylated FOXM1B; (**C**) H1299 cells were transiently transfected with 3 µg HIS-FLAG tagged *FOXM1B* (WT or K463R) and 1 µg HA-*SUMO1* expression plasmids as indicated. After 48 h, cells were harvested and the cell lysates were subjected to Ni^2+^ bead pulldown, followed by anti-FOXM1 and anti-HA immunoblotting. The empty arrows indicate SUMOylated FOXM1B; The solid arrows indicate non-SUMOylated FOXM1B.

To study the biological consequences of FOXM1B SUMOylation, we first aimed to identify the SUMO acceptor site in FOXM1B. SUMOylation typically occurs on lysine residues in a conserved consensus sequence ΨKXE/D. Among the lysine residues of FOXM1B, we identified six potential SUMOylation sites using the SUMOplot analyses program. To facilitate the analysis of FOXM1B SUMOylation, we created HIS-FLAG-tagged mutant forms of FOXM1B in which the acceptor lysines within the SUMOylation motifs were replaced with arginines (Figure 3A). Importantly, these mutant forms of FOXM1B can be readily isolated and distinguished by virtue of the associated FLAG and HIS tags. To determine which lysines in FOXM1B are modified by SUMO1, we probed FOXM1B preparations isolated from MCF7 cells by Ni^2+^ chelate chromatography under denaturing conditions. As can be seen in Figure 3B, a slowly migrating band (about 120–130 kDa) was detected in cells expressing WT and mutant FOXM1Bs except K463R FOXM1B, suggesting that FOXM1B is conjugated by SUMO1 on K463 residue. To further confirm K463 is the major SUMO site for FOXM1B, we co-expressed FOXM1B (WT or K463R) with or without HA-tagged SUMO1 in H1299 cells. As can be seen in Figure 3C, exogenous SUMO1 enhanced SUMOylation on WT FOXM1B but not on K463R FOXM1B. Taken together, these results indicate that FOXM1B can be SUMOylated and K463 is the major SUMO site for FOXM1B.

### 2.4. SUMOylation of FOXM1B at K463 Is Required for FOXM1B’s Transcriptional Activities

**Figure 4:**
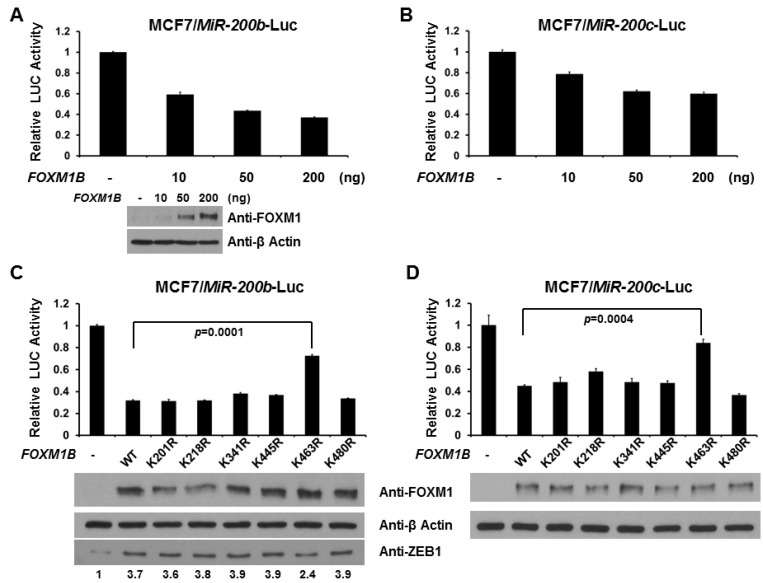
Loss of SUMOylation relieves the repression of MiR-200b/c promoter by FOXM1B. MCF7 cells were co-transfected with different amounts (10, 50, and 200 ng) of *FOXM1B* expression plasmid and a reporter plasmid with *MiR200b* promoter (**A**) or *MiR200c* promoter (**B**); MCF7 cells were transfected, where indicated, with WT or mutant *FOXM1B* expression plasmid and a reporter plasmid with *MiR200b* promoter (**C**) or *MiR200c* promoter (**D**). Luciferase activities were measured 48 h after transfection and normalized with Renilla activity. Relative LUC activity (fold activation) was calculated and plotted. The expression levels of FOXM1B in MCF7 cells from the reporter assays were validated using anti-FOXM1 immunoblotting. The expression levels of ZEB1 in MCF7 cells from the reporter assays with *MiR200b* promoter were validated using anti-ZEB1 immunoblotting. Experiments were performed three times with similar results.

Because FOXM1 is a negative regulator of *MiR-200b/c* gene expression [27] and to gain insight into the role of SUMOylation of FOXM1B, we assessed the effect of this modification on FOXM1B-dependent transcription using a natural *MiR-200b/c* promoter [54,55]. As can be seen in Figure 4A,B, expression of WT FOXM1B leads to a robust dose-dependent reduction in the activity of a *MiR-200b* (Figure 4A) and a *MiR-200c* (Figure 4B) promoter-driven luciferase reporter. As the K463 is the major acceptor site for SUMO in FOXM1B, we next examined whether SUMOylation at K463 is required for FOXM1B activity. As shown in Figure 4C,D, loss of SUMOylation at K463, but not other lysine sites, relieved the reduction by about 50%, suggesting that SUMOylation is required for FOXM1B activity on *MiR-200b/c* promoter regulation. Since MiR-200s have been demonstrated to act as a tumor suppressor by suppressing Zinc-finger enhancing binding transcription factors (ZEB1 and ZEB2) to increase the E-cadherin in cancer cells [56,57], we measured ZEB1 levels from the samples of reporter assays. As shown in Figure 4C, the levels of ZEB1 were increased when WT FOXM1B was expressed. However, loss of SUMOylation at K463, but not other lysine sites, reduces the increase by 43%, further suggesting that SUMO conjugation is required for FOXM1B activity on *MiR-200b/c* promoter regulation.

**Figure 5:**
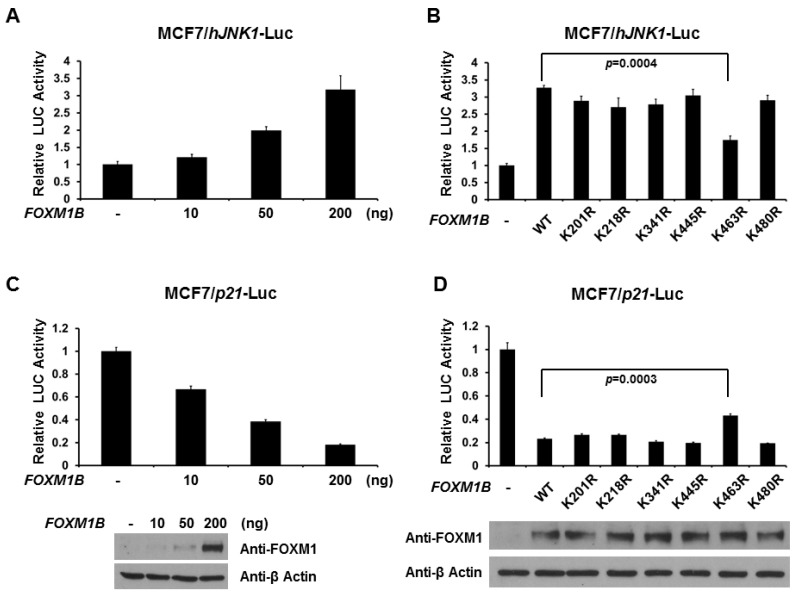
Loss of SUMOylation alters the activities of *JNK1* and *p21* promoters by FOXM1B. MCF7 cells were transfected, where indicated, with different amounts of *FOXM1B* expression plasmid and a reporter plasmid with *JNK1* promoter (**A**) or *p21* promoter (**C**); MCF7 cells were transfected, where indicated, with WT or mutant *FOXM1B* expression plasmid and a reporter plasmid with *JNK1* promoter (**B**) or *p21* promoter (**D**). Luciferase activities were measured 48 h after transfection and normalized with Renilla activity. Relative LUC activity (fold activation) was calculated and plotted. The expression levels of FOXM1B in MCF7 cells from the reporter assays were validated using anti-FOXM1 immunoblotting. Experiments were performed three times with similar results.

FOXM1 is involved in hallmarks of cancer by regulating numerous target genes, including *JNK1*, which is involved in invasion and metastasis, and *p21*, which is involved in cell proliferation. As shown in Figure 5A,C, FOXM1B increases *JNK1* promoter activity but decreases *p21* promoter activity in a dose-dependent manner. This data is consistent with the previous findings [15,18]. Therefore, we next tested whether SUMO modification of FOXM1B alters *JNK1* and *p21* promoter activities. Loss of SUMOylation at K463, but not other lysine sites, decreased FOXM1B-mediated *JNK1* promoter activity by about 50% (Figure 5B). In Figure 5D, loss of SUMOylation at K463 on FOXM1B, but not other lysine sites, relieved the reduction of *p21* promoter activity by about 25%. Taken together, these results provide strong evidence that SUMOylation of FOXM1B at K463 is critical for transcriptional activity of FOXM1B.

### 2.5. Loss of SUMOylation on FOXM1B Reduces Proliferation of MCF7 Cells

**Figure 6:**
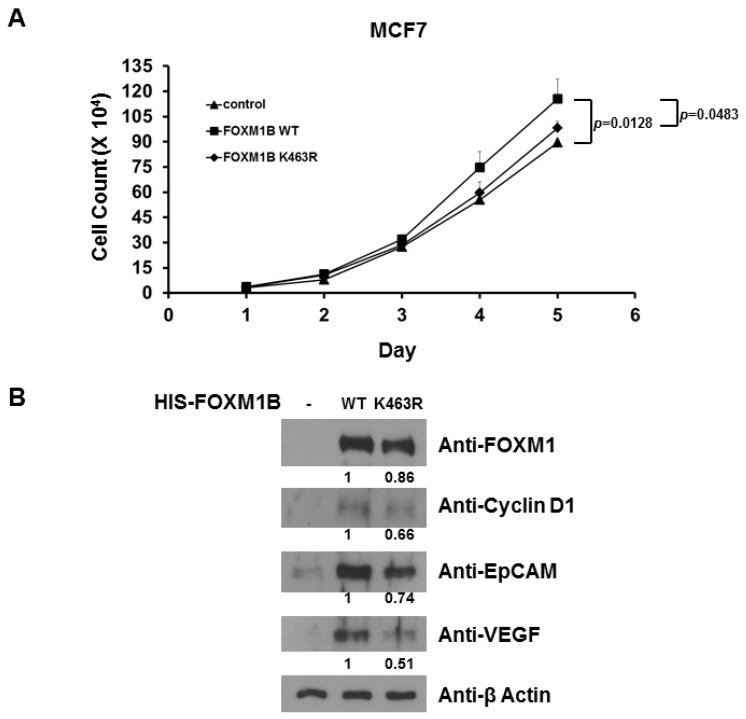
Removal of SUMOylation reduces FOXM1B-mediated cellular proliferation. (**A**) Cell numbers were determined in indicated time after plating of WT FOXM1B-expressed or K463R FOXM1B-expressed MCF7 cells by cell counting assay. On day 5, *p* values were determined; (**B**) On day 5, the expression levels of FOXM1B, Cyclin D1, EpCAM, VEGF (vascular endothelial growth factor), and β-Actin in MCF7 cells were validated using anti-FOXM1, anti-Cyclin D1, anti-EpCAM, anti-VEGF, and anti-β-Actin immunoblotting, respectively. Experiments were performed two times with similar results.

Because FOXM1 is involved in cellular proliferation, we next assessed the potential effect of SUMO modification on FOXM1B in proliferation of MCF7 cells. To evaluate the effect of SUMOylation of FOXM1B on MCF7 cells, recombinant pcDNA3-WT *FOXM1B* and pcDNA3-K463R *FOXM1B* were transfected into MCF7 cells and stably expressed cells were selected. The result showed that cellular growth (Figure 6A) was promoted by the enforced WT FOXM1B over-expression as compared with that of those transfected with empty vector. Interestingly, removal of SUMOylation by the enforced K463R FOXM1B over-expression reduced (compared to WT FOXM1B) cell growth (Figure 4A). The relative protein expression of FOXM1B, Cyclin D1, EpCAM, and VEGF (vascular endothelial growth factor) in FOXM1B-over-expressed MCF7 cells was measured (Figure 6B). These collective data suggest that FOXM1B over-expression plays an important role in promoting cell growth of MCF7 cells and SUMOylation is essential for FOXM1B activity, possibly through regulation of the expression of the several proteins including Cyclin D1, EpCAM, VEGF, and ZEB1.

## 3. Discussion

Regulation of protein function by reversible post-translational modifications such as phosphorylation, is the core principle in biochemistry and molecular and cell biology. Modifications by SUMO proteins have emerged as critical and essential events in a variety of biological processes, including cell cycle regulation, cell death, genomic instability, inflammation, metabolism, transcriptional regulation, and tumor progression. FOXM1B has been shown to be involved in cancer development and progression [10,17,50,51,52,53]; however, the functional significance of SUMO modification for FOXM1B remains to be clarified. In the present work, we demonstrate that the lysine residue, K463, is the major SUMO acceptor site for FOXM1B and SUMOylation is essential, at least in part, for FOXM1B-mediated transcriptional activity.

Alternative splicing of *FOXM1* gene gives rise to three major isoforms of FOXM1: the transcriptionally inactive FOXM1A, and transcriptionally active FOXM1B and FOXM1C variants [49]. FOXM1A, which contains extra A1 and A2 domains from exon Va and exon VIIa, respectively, is transcriptionally inactive due to the presence of an A2 domain which disrupts the transactivation activity. FOXM1C, which contains an A1 domain but not an A2 domain, is transcriptionally active. FOXM1B, which does not have either an A1 or A2 domain, is also transcriptionally active. In the current study, we observed that FOXM1B can be conjugated by SUMO1 and lysine 463 serves as the major SUMO acceptor site for FOXM1B, and loss of SUMOylation on FOXM1B reduces its full capacity on regulating target gene activities. Interestingly, a recent two studies on FOXM1C have shown that several lysine residues on FOXM1C can be SUMOylated and those lysine sites are redundant for SUMO modification and activity of FOXM1C [58,59]. Because SUMOylation is a highly transient, dynamic, and reversible process, the differences in SUMOylation sites identified by the different groups may be attributable to different transforming ability and metastatic potency in cancer development between FOXM1B and FOXM1C and perhaps other upstream regulators that may influence SUMOylation of FOXM1B and FOXM1C. For example, serine 331 (which is missing in FOXM1B) can be phosphorylated by ERK1/2 in FOXM1C [60] and thus, phosphorylation at serine 331 may affect SUMOylation of FOXM1C but not FOXM1B. Therefore, further studies are indeed required to dissect whether phosphorylation status and/or the presence of an A1 domain influences SUMOylation on FOXM1.

FOXM1 has gained much attention and become a subject of intense research in the cancer field. FOXM1 is the relay center in multiple hallmarks of cancer by targeting downstream genes for cancer development, such as proliferation, epithelial-mesenchymal transition (EMT), invasion, and metastasis. A previous study has shown that over-expression of FOXM1 is responsible for EMT phenotype in pancreatic cancer cells, which is in part mediated through the regulation of MiR-200b [27]. MiR-200s have been demonstrated to act as a tumor suppressor by suppressing Zinc-finger enhancing binding transcription factors (ZEB1 and ZEB2) to increase the E-cadherin in cancer cells [56,57]. In the current study, we found that FOXM1B dose-dependently represses promoter activity of *MiR-200b/c* (Figure 4). Most importantly, we found that the repressive effect of FOXM1B on *MiR-200b/c* gene requires SUMOylation at K463 (Figure 4), suggesting the importance of SUMO modification in regulating FOXM1B transcriptional activity. Furthermore, our current study also provided evidence that SUMOylation plays a functional role in FOXM1B’s transcriptional activity in regulating *p21* and *JNK1* promoter activities, which are important for cell proliferation and metastasis, respectively (Figure 5). Since multisite phosphorylations on FOXM1 by various kinases (such as MAPK, CDKs, and PLK1) are essential for FOXM1 activity [45,46,47,48] and the importance of SUMOylation of FOXM1 from our current study and two other previous reports [58,59], there is no doubt that PTMs are critical and essential for FOXM1 activity in various biological processes.

In the current study, we observed that SUMOylation plays a functional role in FOXM1B’s transcriptional activity in regulating *p21* promoter activity. It is worthy to note that the transcriptional activity of p21 is regulated by both p53 and FOXM1, and that FOXM1 is the target of p53. Therefore, the complexity of p21 regulation should be carefully addressed and the dual effect of p53 and FOXM1on p21 activity in cancer cells indeed requires further studies.

In the current study, we observed that FOXM1B is a substrate for SUMO modification and FOXM1B transcriptional activity requires conjugating of SUMO to mediate efficient SUMOylation of FOXM1B at lysine 463. Since SUMOylation of FOXM1B is essential for FOXM1B transcriptional activity, targeting SUMOylation at K463 of FOXM1B provides a suitable therapeutic intervention in addition to the existing FOXM1-based cancer therapies. However, more studies are indeed needed to expand our understanding of how SUMOylation influences FOXM1 activity in cancers. Collectively, our results not only extend the conclusion that FOXM1 is involved in the hallmarks of cancer processes but also provide the novel mechanism of how SUMOylatgion regulates FOXM1B activity.

## 4. Experimental Section

### 4.1. Reagents

All cell culture reagents were purchased from Life Technologies (Carlsbad, CA, USA). Protein A and protein G magnetic beads were purchased from Fisher Scientific (Pittsburgh, PA, USA). Antibodies against FOXM1, HA, HIS, Cyclin D1, EpCAM, VEGF,and β-Actin (Santa Cruz Biotechnology Inc., Santa Cruz, CA, USA), SUMO1 (Active motif, Carsbad, CA, USA), and FLAG (Sigma, St. Louis, MO, USA) were purchased commercially. Luciferase activity was measured using the Dual Luciferase Assay System (Promega, Madison, WI, USA). Ni–NTA agarose was purchased from QIAGEN (Valencia, CA, USA).

### 4.2. DNA Constructs

Human HIS-FLAG-*FOXM1C* cDNA was polymerase chain reaction (PCR)-amplified and ligated into the *Hin*dIII and *Bam*HI sites of pcDNA3(+) to create pcDNA3-HIS-FLAG-*FOXM1C* expression plasmid. Human HIS-FLAG-*FOXM1B* plasmid was constructed by removal the A1 domain of *FOXM1C* plasmid.Human HIS-*FOXM1B* and FLAG-*FOXM1B* plasmids were constructed by removal of the HIS tag and FLAG tag of HIS-FLAG-*FOXM1B* plasmid, respectively. HA-*SUMO1*-pcDNA3 and HA-*SENP2*-pcDNA6 plasmidswere previously established in our laboratory as described in Wang *et al.* [30]. HIS-*SUMO1*-pcDNA3 and FLAG-*SENP2*-pcDNA6 plasmids were generated by PCR-based mutagenesis (QuikChange Lightning site-directed mutagenesis kit, Strategene, La Jolla, CA, USA). FLAG-PIASy expression plasmid was created previously in our laboratory [30]. *MiR-200b/a/429* and *MiR-200c/141* promoter luciferase plasmids (both in pGL3) were kindly provided by Dr. Tewari (Fred Hutchison Cancer Research Center, Seattle, Washington, DC, USA). Human *JNK1* promoter luciferase plasmid (h*JNK1*-LUC, 1.3 kb upstream the transcription start site) was kindly provided by Drs. Costa/Raychauhuri (University of Illinois, Chicago, IL, USA). *p21* promoter luciferase plasmid (*p21*-LUC) was previously described in Liu *et al.* [61]. All constructs were verified by nucleotide sequencing.

### 4.3. Cell Culture and Transfection

MCF7 and H1299 cells were purchased from the American Type Culture Collection. MCF7 and H1299 cells were maintained in Dulbecco’s modified Eagle’s medium (DMEM) in the presence of 10% fetal bovine serum and antibiotics (GIBCO/Life Technologies, Grand Island, NY, USA) in humidified air containing 5% CO_2_, at 37 °C. After incubation, the cells were transfected using Fugene HD Transfection Reagent (Roche, Madison, WI, USA). Approximately 45−48 h after transfection, the cells were harvested. Luciferase activity was measured and normalized with Renilla activity. All experiments were performed three times in triplicate.

### 4.4. Immunoprecipitation Assay

MCF7 or H1299 cells (2 × 10^6^) were seeded onto 10-cm plates. Twenty-four hours after transient transfection, cells were harvested and lysed in lysis buffer (40 mM HEPES, 120 mM sodium chloride, 10 mM sodium pyrophosphate, 10 mM sodium glycerophosphate, 1 mM EDTA, 50 mM sodium fluoride, 0.5 mM sodium orthovanadate, 1% Triton X-100) containing protease inhibitor cocktail (Sigma, St. Louis, MO, USA), followed by rotation for 1 h at 4 °C to solubilize proteins. Soluble proteins were collected and immunoprecipitated with the indicated antibody overnight. Protein A or G magnetic beads were added to protein lysates for 2 h in the cold room. Beads were separated from lysate solution by magnetic force (in a magnetic separation rack) and washed at least three times with lysis buffer. For Ni^2+^-bead pull-down assays, Ni^2+^-NTA agarose was used to precipitate HIS-tagged FOXM1B from cell lysates. Proteins were eluted by boiling in 50 µL of 2× Laemmlisample buffer, resolved by 8%–10% SDS-PAGE, and processed for immunoblotting as described below.

### 4.5. Immunoblotting

Protein lysates were allowed to rotate at 4 °C for 30 min, and protein contents ofthe high-speed supernatant were determined using the BCA™ Protein Assay kit assay (Pierce/Thermo Scientific, Rockford, IL, USA). Equivalent quantities of protein (25–40 µg) were resolved on polyacrylamide-SDS gels, transferred to nitrocellulose membrane (Bio-Rad, Hercules, CA, USA), and immunoblotted with specific antibodies. Results were visualized using the Supersignal West Dura Extended Duration Substrate kit (Pierce Chemical Co., Rockford, IL, USA). Band intensity was quantified by ImageJ program (National Institutes of Health (NIH), Bethesda, MD, USA).

### 4.6. In Vivo SUMOylation Assays

The *in vivo* SUMOylation assay was carried out as previously described [33]. Briefly, MCF7 or H1299 cells (2 × 10^6^) were seeded in 10 cm plates and 24 h later were transfected with indicated HIS-FLAG-*FOXM1B* expression vectors. After 48 h, cells were harvested in 700 µL lysis buffer (500 mM NaCl, 10 mM imidazole, 45 mM Na_2_HPO_4_, 5 mM Na_2_H2PO_4_, 8 M urea, pH 8.0) containing complete protease inhibitors without EDTA (1 tablet/10 mL; Roche, Madison, WI, USA) and sonicated. Lysates were cleared and incubated with 100 µL of 50% Ni^2+^–NTA agarose (QIAGEN, Valencia, CA, USA) at room temperature for 60 min on a rotator. The resin was washed 3 times in wash buffer 1 (400 mM NaCl, 10 mM imidazole, 17.6 mM Na_2_HPO_4_, 32.4 mM Na_2_H_2_PO_4_, 8 M urea, pH 6.75), washed 3 times in wash buffer 2 (150 mM NaCl, 10 mM imidazole, 17.6 mM Na_2_HPO_4_, 32.4 mM Na_2_H_2_PO_4_, pH 6.75). Samples were resuspended in 2× EDTA SDS-PAGE sample buffer. Samples (20 µL) were resolved by 8%–10% SDS-PAGE and processed forimmunoblotting using anti-FOXM1 or anti-HA (for SUMO1) primary antibody. Images were captured in a Kodak Image Station 440 CF using Super Signal West Fem to substrates (Thermo scientific/Pierce, Rockford, IL, USA).

### 4.7. Cell Proliferation Assay

MCF7 cells stably expressed WT or K463R FOXM1B were seeded in a six-well plate at a concentration of 5 × 10^3^ per well. At 0, 1, 2, 3, 4, and 5 days in culture, cell proliferation was measured by trypan blue exclusion using a microscope.

### 4.8. Statistical Analysis

Statistical analyses were performed using the Student’s *t* test or a one-way ANOVA (analysis of variance) when more than two groups were compared. After the ANOVA analysis, the *post hoc* multiple comparisons were performed by using Tukey honestly significant difference (HSD) test to determine the statistical difference from each other among subgroups. For each test, *p* values less than 0.05 were considered significant.

## 5. Conclusions

In summary, this investigation has demonstrated that lysine 463 is the main SUMO site for FOXM1B and SUMOylation serves as an important regulator on the transcriptional activity of FOXM1B. Our study also adds a new layer of information to the previous understanding of how FOXM1B functions to regulate cell cycle progression and tumor initiation, promotion, and metastasis.

## Abbreviations

FOXM1, forkhead box protein M1; JNK1, mitogen-activated protein kinase 8; SENP2, sentrin-pecific protease 2; SUMO, small ubiquitin-like modifier.

## References

[1] Raychaudhuri P., Park H.J. (2011). FoxM1: A master regulator of tumor metastasis. Cancer Res..

[2] Chen X., Müller G.A., Quaas M., Fischer M., Han N., Stutchbury B., Sharrocks A.D., Engeland K. (2013). The forkhead transcription factor FOXM1 controls cell cycle-dependent gene expression through an atypical chromatin binding mechanism. Mol. Cell. Biol..

[3] Nakamura S., Hirano I., Okinaka K., Takemura T., Yokota D., Ono T., Shigeno K., Shibata K., Fujisawa S., Ohnishi K. (2010). The FOXM1 transcriptional factor promotes the proliferation of leukemia cells through modulation of cell cycle progression in acute myeloid leukemia. Carcinogenesis.

[4] Petrovic V., Costa R.H., Lau L.F., Raychaudhuri P., Tyner A.L. (2008). FoxM1 regulates growth factor-induced expression of kinase-interacting stathmin (KIS) to promote cell cycle progression. J. Biol. Chem..

[5] Qian J., Luo Y., Gu X., Zhan W., Wang X. (2013). Twist1 promotes gastric cancer cell proliferation through up-regulation of *FoxM1*. PLoS One.

[6] Qu K., Xu X., Liu C., Wu Q., Wei J., Meng F., Zhou L., Wang Z., Lei L., Liu P. (2013). Negative regulation of transcription factor *FoxM1* by p53 enhances oxaliplatin-induced senescence in hepatocellular carcinoma. Cancer Lett..

[7] Pandit B., Halasi M., Gartel A.L. (2009). p53 negatively regulates expression of *FoxM1*. Cell Cycle.

[8] Barsotti A.M., Prives C. (2009). Pro-proliferative *FoxM1* is a target of p53-mediated repression. Oncogene.

[9] Wierstra I., Alves J. (2006). Transcription factor FOXM1c is repressed by RB and activated by cyclin D1/Cdk4. Biol. Chem..

[10] Kalinichenko V.V., Major M.L., Wang X., Petrovic V., Kuechle J., Yoder H.M., Dennewitz M.B., Shin B., Datta A., Raychaudhuri P. (2004). *Foxm1b* transcription factor is essential for development of hepatocellular carcinomas and is negatively regulated by the p19ARF tumor suppressor. Genes Dev..

[11] Halasi M., Gartel A.L. (2009). A novel mode of *FoxM1* regulation: Positive auto-regulatory loop. Cell Cycle.

[12] Mirza M.K., Sun Y., Zhao Y.D., Potula H.H., Frey R.S., Vogel S.M., Malik A.B., Zhao Y.Y. (2010). *FoxM1* regulates re-annealing of endothelial adherens junctions through transcriptional control of β-catenin expression. J. Exp. Med..

[13] Bowman A., Nusse R. (2011). Location, location, location: *FoxM1* mediates β-catenin nuclear translocation and promotes gliomatumorigenesis. Cancer Cell.

[14] Zhang N., Wei P., Gong A., Chiu W.T., Lee H.T., Colman H., Huang H., Xue J., Liu M., Wang Y. (2011). *FoxM1* promotes β-catenin nuclear localization and controls Wnt target-gene expression and glioma tumorigenesis. Cancer Cell.

[15] Wang X., Hung N.J., Costa R.H. (2001). Earlier expression of the transcription factor HFH-11B diminishes induction of p21 (CIP1/WAF1) levels and accelerates mouse hepatocyte entry into *S*-phase following carbon tetrachloride liver injury. Hepatology.

[16] Zhang Y., Zhang N., Dai B., Liu M., Sawaya R., Xie K., Huang S. (2008). *FoxM1B* transcriptionally regulates vascular endothelial growth factor expression and promotes the angiogenesis and growth of glioma cells. Cancer Res..

[17] Dai B., Kang S.H., Gong W., Liu M., Aldape K.D., Sawaya R., Huang S. (2007). Aberrant FoxM1B expression increases matrix metalloproteinase-2 transcription and enhances the invasion of glioma cells. Oncogene.

[18] Wang I.C., Chen Y.J., Hughes D.E., Ackerson T., Major M.L., Kalinichenko V.V., Costa R.H., Raychaudhuri P., Tyner A.L., Lau L.F. (2008). *FoxM1* regulates transcription of JNK1 to promote the G_1_/S transition and tumor cell invasiveness. J. Biol. Chem..

[19] Waseem A., Ali M., Odell E.W., Fortune F., Teh M.T. (2010). Downstream targets of FOXM1: CEP55 and HELLS are cancer progression markers of head and neck squamous cell carcinoma. Oral Oncol..

[20] Wang I.C., Chen Y.J., Hughes D., Petrovic V., Major M.L., Park H.J., Tan Y., Ackerson T., Costa R.H. (2005). Forkhead box M1 regulates the transcriptional network of genes essential for mitotic progression and genes encoding the SCF (Skp2–Cks1) ubiquitin ligase. Mol. Cell. Biol..

[21] Madureira P.A., Varshochi R., Constantinidou D., Francis R.E., Coombes R.C., Yao K.M., Lam E.W. (2006). The Forkhead box M1 protein regulates the transcription of the estrogen receptor alpha in breast cancer cells. J. Biol. Chem..

[22] Halasi M., Gartel A.L. (2013). FOX (M1) news—It is cancer. Mol. Cancer Ther..

[23] Xu N., Jia D., Chen W., Wang H., Liu F., Ge H., Zhu X., Song Y., Zhang X., Zhang D. (2013). *FoxM1* is associated with poor prognosis of non-small cell lung cancer patients through promoting tumor metastasis. PLoS One.

[24] Wu X.R., Chen Y.H., Liu D.M., Sha J.J., Xuan H.Q., Bo J.J., Huang Y.R. (2013). Increased expression of forkhead box M1 protein is associated with poor prognosis in clear cell renal cell carcinoma. Med. Oncol..

[25] Sun H.C., Li M., Lu J.L., Yan D.W., Zhou C.Z., Fan J.W., Qin X.B., Tang H.M., Peng Z.H. (2011). Over-expression of Forkhead box M1 protein associates with aggressive tumor features and poor prognosis of hepatocellular carcinoma. Oncol. Rep..

[26] Kong X., Li L., Li Z., Le X., Huang C., Jia Z., Cui J., Huang S., Wang L., Xie K. (2013). Dysregulated expression of FOXM1 isoforms drives progression of pancreatic cancer. Cancer Res..

[27] Bao B., Wang Z., Ali S., Kong D., Banerjee S., Ahmad A., Li Y., Azmi A.S., Miele L., Sarkar F.H. (2011). Over-expression of *FoxM1* leads to epithelial-mesenchymal transition and cancer stem cell phenotype in pancreatic cancer cells. J. Cell Biochem..

[28] Gong L., Ji W.K., Hu X.H., Hu W.F., Tang X.C., Huang Z.X., Li L., Liu M., Xiang S.H., Wu E. (2014). Sumoylation differentially regulates Sp1 to control cell differentiation. Proc. Natl. Acad. Sci. USA.

[29] Lee G.Y., Jang H., Lee J.H., Huh J.Y., Choi S., Chung J., Kim J.B. (2014). PIASy-mediated sumoylation of SREBP1c regulates hepatic lipid metabolism upon fasting signaling. Mol. Cell. Biol..

[30] Wang C.M., Brennan V.C., Gutierrez N.M., Wang X., Wang L., Yang W.H. (2013). SUMOylation of ATF3 alters its transcriptional activity on regulation of *TP53* gene. J. Cell Biochem..

[31] Nepveu-Traversy M.É., Berthoux L. (2014). The conserved sumoylation consensus site in TRIM5α modulates its immune activation functions. Virus Res..

[32] Lee P.C., Taylor-Jaffe K.M., Nordin K.M., Prasad M.S., Lander R.M., LaBonne C. (2012). SUMOylated SoxE factors recruit Grg4 and function as transcriptional repressors in the neural crest. J. Cell Biol..

[33] Yang W.H., Heaton J.H., Brevig H., Mukherjee S., Iñiguez-Lluhí J.A., Hammer G.D. (2009). SUMOylation inhibits SF-1 activity by reducing CDK7-mediated serine 203 phosphorylation. Mol. Cell. Biol..

[34] Qin Y., Bao H., Pan Y., Yin M., Liu Y., Wu S., Li H. (2014). SUMOylation alterations are associated with multidrug resistance in hepato cellular carcinoma. Mol. Med. Rep..

[35] Gareau J.R., Lima C.D. (2010). The SUMO pathway: Emerging mechanisms that shape specificity, conjugation and recognition. Nat. Rev. Mol. Cell. Biol..

[36] Aukrust I., Bjørkhaug L., Negahdar M., Molnes J., Johansson B.B., Müller Y., Haas W., Gygi S.P., Søvik O., Flatmark T. (2013). SUMOylation of pancreatic glucokinase regulates its cellular stability and activity. J. Biol. Chem..

[37] Gong Z., Brackertz M., Renkawitz R. (2006). SUMO modification enhances p66-mediated transcriptional repression of the Mi-2/NuRD complex. Mol. Cell. Biol..

[38] Rytinki M.M., Palvimo J.J. (2008). SUMOylation modulates the transcription repressor function of RIP140. J. Biol. Chem..

[39] Abed M., Barry K.C., Kenyagin D., Koltun B., Phippen T.M., Delrow J.J., Parkhurst S.M., Orian A. (2011). Degringolade, a SUMO-targeted ubiquitin ligase, inhibits Hairy/Groucho-mediated repression. EMBO J..

[40] Duverger O., Chen S.X., Lee D., Li T., Chock P.B., Morasso M.I. (2011). SUMOylation of DLX3 by SUMO1 promotes its transcriptional activity. J. Cell Biochem..

[41] Rodriguez M.S., Desterro J.M., Lain S., Midgley C.A., Lane D.P., Hay R.T. (1999). SUMO-1 modification activates the transcriptional response of p53. EMBO J..

[42] Guo Y., Yang M.C., Weissler J.C., Yang Y.S. (2008). Modulation of PLAGL2 transactivation activity by Ubc9 co-activation not SUMOylation. Biochem. Biophys. Res. Commun..

[43] Ihara M., Stein P., Schultz R.M. (2008). UBE2I (UBC9), a SUMO-conjugating enzyme, localizes to nuclear speckles and stimulates transcription in mouse oocytes. Biol. Reprod..

[44] Arora T., Liu B., He H., Kim J., Murphy T.L., Murphy K.M., Modlin R.L., Shuai K. (2003). PIASx is a transcriptional co-repressor of signal transducer and activator of transcription 4. J. Biol. Chem..

[45] Joshi K., Banasavadi-Siddegowda Y., Mo X., Kim S.H., Mao P., Kig C., Nardini D., Sobol R.W., Chow L.M., Kornblum H.I. (2013). MELK-dependent *FOXM1* phosphorylation is essential for proliferation of glioma stem cells. Stem Cells.

[46] Chen Y.J., Dominguez-Brauer C., Wang Z., Asara J.M., Costa R.H., Tyner A.L., Lau L.F., Raychaudhuri P. (2009). A conserved phosphorylation site within the forkhead domain of *FoxM1B* is required for its activation by cyclin-CDK1. J. Biol. Chem..

[47] Fu Z., Malureanu L., Huang J., Wang W., Li H., van Deursen J.M., Tindall D.J., Chen J. (2008). Plk1-dependent phosphorylation of *FoxM1* regulates a transcriptional programme required for mitotic progression. Nat. Cell Biol..

[48] Major M.L., Lepe R., Costa R.H. (2004). Forkhead box M1B transcriptional activity requires binding of Cdk-cyclin complexes for phosphorylation-dependent recruitment of p300/CBP coactivators. Mol. Cell. Biol..

[49] Laoukili J., Stahl M., Medema R.H. (2007). *FoxM1*: At the crossroads of ageing and cancer. Biochim. Biophys. Acta.

[50] Lam A.K., Ngan A.W., Leung M.H., Kwok D.C., Liu V.W., Chan D.W., Leung W.Y., Yao K.M. (2013). *FOXM1b*, which is present at elevated levels in cancer cells, has a greater transforming potential than *FOXM1c*. Front. Oncol..

[51] Liu M., Dai B., Kang S.H., Ban K., Huang F.J., Lang F.F., Aldape K.D., Xie T.X., Pelloski C.E., Xie K. (2006). FoxM1B is over-expressed in human glioblastomas and critically regulates the tumorigenicity of glioma cells. Cancer Res..

[52] Kalinina O.A., Kalinin S.A., Polack E.W., Mikaelian I., Panda S., Costa R.H., Adami G.R. (2003). Sustained hepatic expression of *FoxM1B* in transgenic mice has minimal effects on hepatocellular carcinoma development but increases cell proliferation rates in preneoplastic and early neoplastic lesions. Oncogene.

[53] Park H.J., Gusarova G., Wang Z., Carr J.R., Li J., Kim K.H., Qiu J., Park Y.D., Williamson P.R., Hay N. (2011). Deregulation of *FoxM1b* leads to tumour metastasis. EMBO Mol. Med..

[54] Knouf E.C., Garg K., Arroyo J.D., Correa Y., Sarkar D., Parkin R.K., Wurz K., O’Briant K.C., Godwin A.K., Urban N.D. (2012). An integrative genomic approach identifies p73 and p63 as activators of miR-200 microRNA family transcription. Nucleic Acids Res..

[55] Bendoraite A., Knouf E.C., Garg K.S., Parkin R.K., Kroh E.M., O’Briant K.C., Ventura A.P., Godwin A.K., Karlan B.Y., Drescher C.W. (2010). Regulation of miR-200 family microRNAs and ZEB transcription factors in ovarian cancer: Evidence supporting a mesothelial-to-epithelial transition. Gynecol. Oncol..

[56] Gregory P.A., Bert A.G., Paterson E.L., Barry S.C., Tsykin A., Farshid G., Vadas M.A., Khew-Goodall Y., Goodall G.J. (2008). The miR-200family and miR-205 regulate epithelial to mesenchymal transition by targeting ZEB1 and SIP1. Nat. Cell Biol..

[57] Brabletz S., Brabletz T. (2010). The ZEB/miR-200 feedback loop—A motor of cellular plasticity in development and cancer?. EMBO Rep..

[58] Myatt S.S., Kongsema M., Man C.W., Kelly D.J., Gomes A.R., Khongkow P., Karunarathna U., Zona S., Langer J.K., Dunsby C.W. (2014). SUMOylation inhibits FOXM1 activity and delays mitotic transition. Oncogene.

[59] Schimmel J., Eifler K., Sigurðsson J.O., Cuijpers S.A., Hendriks I.A., Verlaan-de Vries M., Kelstrup C.D, Francavilla C., Medema R.H., Olsen J.V. (2014). Uncovering SUMOylation dynamics during cell-cycle progression reveals *FoxM1* as a key mitotic SUMO target protein. Mol. Cell.

[60] Dephoure N., Zhou C., Villén J., Beausoleil S.A., Bakalarski C.E., Elledge S.J., Gygi S.P. (2008). A quantitative atlas of mitotic phosphorylation. Proc. Natl. Acad. Sci. USA.

[61] Liu R., Wang L., Chen G., Katoh H., Chen C., Liu Y., Zheng P. (2009). FOXP3 up-regulates p21 expression by site-specific inhibition of histone deacetylase 2/histone deacetylase 4 association to the locus. Cancer Res..

